# Engaging in culturally responsive and antiracism research and programs for Black Canadian communities

**DOI:** 10.24095/hpcdp.45.4.01

**Published:** 2025-04

**Authors:** Jude Mary Cnat, Asha Lofters, Josephine Etowa

**Affiliations:** 1 School of Psychology, University of Ottawa, Ottawa, Ontario, Canada; 2 Interdisciplinary Centre for Black Health, University of Ottawa, Ottawa, Ontario, Canada; 3 Dalla Lana School of Public Health, University of Toronto, Toronto, Ontario, Canada; 4 School of Nursing, University of Ottawa, Ottawa, Ontario, Canada

## Introduction

In 2021, Black people represented 4.3% of the general population in Canada.^1^ Compared to other racial or ethnic groups, they experienced the fastest growth over the past 20 years, with their population doubling from 662210 Black individuals in 2001 to 1.5 million in 2021.^1^-^3^ Originating from 182 countries, compared to 230birthplaces for the general population, Black communities are culturally diverse. They include individuals whose ancestors have been in Canada for centuries, such as those in Nova Scotia, as well as children and grandchildren of immigrants and recent immigrants from countries such as Jamaica, Haiti, Nigeria and Ethiopia.^1^

According to the latest census data, 4 in 10 Black Canadians were born in Canada, while more than three in four Black youth under 14 years old (77% of Black youth born in Canada out of the general population of Black youth aged under 14 years old) were also born in Canada.^1^ Compared to the general population, 2021 census data show that Black communities are significantly younger.^1^ Overall, 42% of Black individuals are under 25 years of age, compared to 28% in the general population. About 26% of Black people in Canada are under 15 years old, compared to 16% in the general population. Furthermore, only 6% of Black individuals are 65 years and older, compared to 18% in the general population.^1^

These data reflect the unique demographics and dynamics that influence social, economic and educational spheres. They represent a young and dynamic profile, contrasting with the aging observed in the general population. However, Black individuals, regardless of their birthplace, face various inequities that hinder both their physical and mental health, leading to limited access to healthcare services, and to their receiving lower-quality care compared to White communities.^4^,^5^

## Understanding racial health disparities in Black communities in Canada

The physical and mental health disparities in Black communities in Canada reflect a complex combination of systemic, social, economic, educational and individual determinants that structure their experience within society.^6^-^8^ Studies conducted over the last decade have shown that these disparities stem from five main challenges: (1) systemic and institutional racism faced by Black individuals in different areas of society, hindering their physical and mental health;^9^ (2) a lack of reliable data preventing the development of evidence-based health policies for Black communities;^10^ (3) insufficient training of healthcare professionals, limiting their ability to provide culturally responsive and antiracist care;^11^ (4) the absence of political strategies to identify, examine and reduce racial inequalities in healthcare;^4^,^5^,^12^ and (5) a lack of funding for health research on Black populations.

These inequalities are exacerbated by social determinants of health such as adversity, economic insecurity, precarious employment, income inequality and poverty, food insecurity, inadequate housing, exclusion and experience of racial discrimination, including in healthcare services.^13^ Observed disparities include insufficient access to chronic disease screening (e.g. diabetes, hypertension, cancer^14^), limited disease management and self-care. 

In the area of mental health, studies published since 2021 have documented the prevalence of and factors associated with depression, anxiety, posttraumatic stress disorder (PTSD), psychosomatic symptoms, alcohol and substance use, suicidal ideation and more.^15^-^20^ They have shown that the prevalence of severe symptoms of anxiety, depression, PTSD and other disorders is significantly higher in Black communities. They also indicate that daily racial discrimination, major racial discrimination in various areas of society (e.g. healthcare, education, interactions with police), racial microaggressions and internalized racism are the main explanatory factors for poor mental health in Black populations in Canada. Furthermore, studies have shown that Black individuals have limited access to mental health care and a heightened distrust of available professionals and services.^21^,^22^

These inequalities have serious consequences, leading to a reduced quality of life, an increased burden on Black communities and an amplification of socioeconomic inequities. Moreover, the lack of evidence and representative studies on the health of Black people in Canada limits policy makers’ ability to create effective policies to reduce disparities and promote health equity. This gap also reduces the ability to train students and healthcare professionals on racial issues and to provide culturally responsive and antiracist healthcare. Notably, systematic reviews conducted in recent years have shown disparities in cancer screening and care, venous thromboembolism, psychosis, mental health issues and health behaviours, including vaccine hesitancy among Black youth.^14^,^21^,^23^-^25^

The COVID-19 pandemic exacerbated racial health inequities and highlighted the absence of Black individuals in public health decision-making. Not only were Black people disproportionately affected in terms of infections and mortality, but they were also less likely to get vaccinated against COVID-19,^26^,^27^ hence the need for this initiative to give voice to research documenting various health needs in Black communities by engaging them in culturally responsive and antiracist ways.

## A special issue on engaging in culturally responsive research and programs for Black communities in Canada

This special issue presents five articles, including three evidence synthesis articles, one study protocol with methodological reflections about how to engage with Black youth about their mental health needs, and a qualitative research article based on an empirical evaluation of federal funding for mental health initiatives for Black people in Canada. 

The first article, by Jamieson and colleagues, is a scoping review that explores the lack of data on racial health inequalities in Canada, highlighting challenges to and opportunities for improving the measurement and monitoring of health disparities among racialized populations, particularly Black Canadians.^28^ Using a multistep methodology, the authors examined survey methods used in Canada and comparable countries (United States, United Kingdom, Australia and New Zealand) to identify promising practices for improving sampling strategies and data collection. The results show significant gaps in health surveys in Canada, which do not use targeted sampling strategies, such as utilizing racial or ethnic concentrations at the geographic level to increase the representation of racialized groups. In contrast, similar countries adopt these approaches to produce larger and more representative datasets. The authors recommend the adoption of targeted sampling, oversampling and predictive modelling methods in Canada to better include racialized populations. They also emphasize the importance of integrating race data into administrative databases for more effective monitoring of inequalities. 

A second scoping review, by Mombo et al. examines methods of collecting, analyzing and disseminating data on the health and social determinants of Black communities in Quebec.^29^ Although Black populations in Quebec represent over a quarter of Canada’s Black population, little research on this population exists, including data on the impacts of the COVID-19 pandemic. This review of 43 studies highlights the challenges and strategies for data collection and analysis, focussing on the need for a better understanding of the lived realities of Black populations in Quebec. The studies cover four sectors: health, social services, education and employment. 

The third article, by Yusuf et al., is a rapid review that examines culturally and structurally competent approaches to health research with Black communities in Canada’s Atlantic provinces.^30^ Although Black populations have resided in this region since the late 17th century, they face significant inequalities, including the highest rates of child poverty in the country. Forty-seven studies were included, highlighting the impact of racism, the importance of community engagement and the adoption of participatory research frameworks as culturally appropriate practices. 

The fourth article presented in this special issue, by Salami and colleagues, explores how to engage Black youth in research, using a culturally sensitive participatory action research approach to examine their mental health needs.^31^ The study involved two phases: individual interviews with 30youths and monthly conversation cafs with 99 youths over a four-month period. Participants were recruited through community networks in Alberta, promoting youth empowerment and collaboration. The findings were shared with stakeholders, highlighting culturally relevant strategies to improve access to mental health services. 

The final study, by Salami et al., examines lessons learned from the Public Health Agency of Canada’s Mental Health of Black Canadians Fund, created in 2018 to address mental health inequalities among Black Canadians.^32^ This analysis of annual and final reports from 15 projects, as well as interviews with representatives from nine organizations that received funding, reveals three main themes: success factors, challenges encountered and lessons learned. Success factors include honoraria and incentives, participatory action research and Black leadership. Challenges include delays in obtaining funds, the impacts of the COVID-19 pandemic and maintaining partnerships. 

## Conclusion

This special issue of Health Promotion and *Chronic Disease Prevention in Canada* is of vital importance in highlighting health inequalities among Black communities in Canada and offering solutions to address them. It is even more important in a changing North America, where programs related to equity, diversity, inclusion and accessibility, as well as those aimed at combating anti-Black racism, are increasingly under attack and face an uncertain future. It showcases innovative research on data collection, community engagement and culturally and structurally competent research approaches. The focus is on the need to include more perspectives from Black communities in public health decision-making, improve the training of healthcare professionals and enhance Black representation in funding organizations to better support equitable health policies. This issue represents a crucial step toward addressing current challenges and promoting health equity for racialized populations in Canada.

## Funding

None.

## Conflicts of interest

The authors declare there are no conflicts of interest.

## Statement

The content and views expressed in this article are those of the authors and do not necessarily reflect those of the Government of Canada.
